# A two-system theory of sensory-evoked brain responses

**DOI:** 10.1093/brain/awaf402

**Published:** 2025-10-23

**Authors:** Richard Somervail, Sofija Perovic, Rory John Bufacchi, Roberto Caminiti, Gian Domenico Iannetti

**Affiliations:** Neuroscience & Behaviour Laboratory, Italian Institute of Technology (IIT), Rome 00161, Italy; Department of Neuroscience, Physiology & Pharmacology, University College London (UCL), London WC1E 6BT, UK; Translational and Computational Neuroscience Unit, Department of Psychology, Manchester Metropolitan University, Manchester M15 6GX, UK; Neuroscience & Behaviour Laboratory, Italian Institute of Technology (IIT), Rome 00161, Italy; Department of Psychology, Sapienza University of Rome, Rome 00161, Italy; Neuroscience & Behaviour Laboratory, Italian Institute of Technology (IIT), Rome 00161, Italy; International Center for Primate Brain Research (ICPBR), Center for Excellence in Brain Science and Intelligence Technology, Chinese Academy of Sciences, Shanghai 201602, China; Neuroscience & Behaviour Laboratory, Italian Institute of Technology (IIT), Rome 00161, Italy; Neuroscience & Behaviour Laboratory, Italian Institute of Technology (IIT), Rome 00161, Italy; Department of Neuroscience, Physiology & Pharmacology, University College London (UCL), London WC1E 6BT, UK

**Keywords:** EEG, fMRI, saliency, surprise, thalamus, sensory systems

## Abstract

Sudden and isolated sensory stimuli (SISS) likely signal environmental events demanding immediate behavioural responses. These stimuli—which are widely and persistently used in both basic and clinical neuroscience to explore sensory processing and perception—also trigger some of the largest and most widespread electrocortical responses in the awake mammalian brain. These responses are often interpreted as reflecting either modality-specific sensory processing mediated by high-fidelity ‘lemniscal’ thalamocortical pathways to primary sensory cortices, cortico-cortical connections or motor activity.

Here we contend that these interpretations are unjustified. We first describe evidence that the electrocortical responses elicited by the SISS used in systems and cognitive neuroscience are strongly contributed to by non–modality-specific processes mediated by diffuse ‘extralemniscal’ thalamocortical projections. In human EEG, this contribution is reflected in the scalp vertex potential. We then discuss the implications of this ‘two-system’ theory for basic and clinical neuroscience studies, including the neural correlates of consciousness, where widespread responses to sudden, isolated or rare stimuli—often interpreted as signatures of awareness—may instead reflect extralemniscal activity. We conclude by suggesting a mechanism through which transient extralemniscal responses affect ongoing brain activity and promote swift reactions to sudden environmental changes.

## Teaser

Surprising sensory stimuli—such as loud beeps—are often used to study how the brain processes sensation. Somervail *et al.* show that these stimuli activate much more of the brain than previously thought, through a long-overlooked pathway called the extralemniscal system, with implications for basic and clinical neuroscience.

## Transient sensory brain responses reflect the activity of two parallel thalamocortical systems

To survive in a dynamic world, an organism must infer its state, a process that implies making predictions about sensory inputs.^1^ Surprising environmental events reflect the violation of these predictions and often require behavioural reactions. Perhaps the simplest example of such an event is a sudden and isolated change of stimulus energy (SISS; sudden and isolated sensory stimulus), which breaks the expectation that the environment will remain stable in the short term.^2-4^

**Figure 1:**
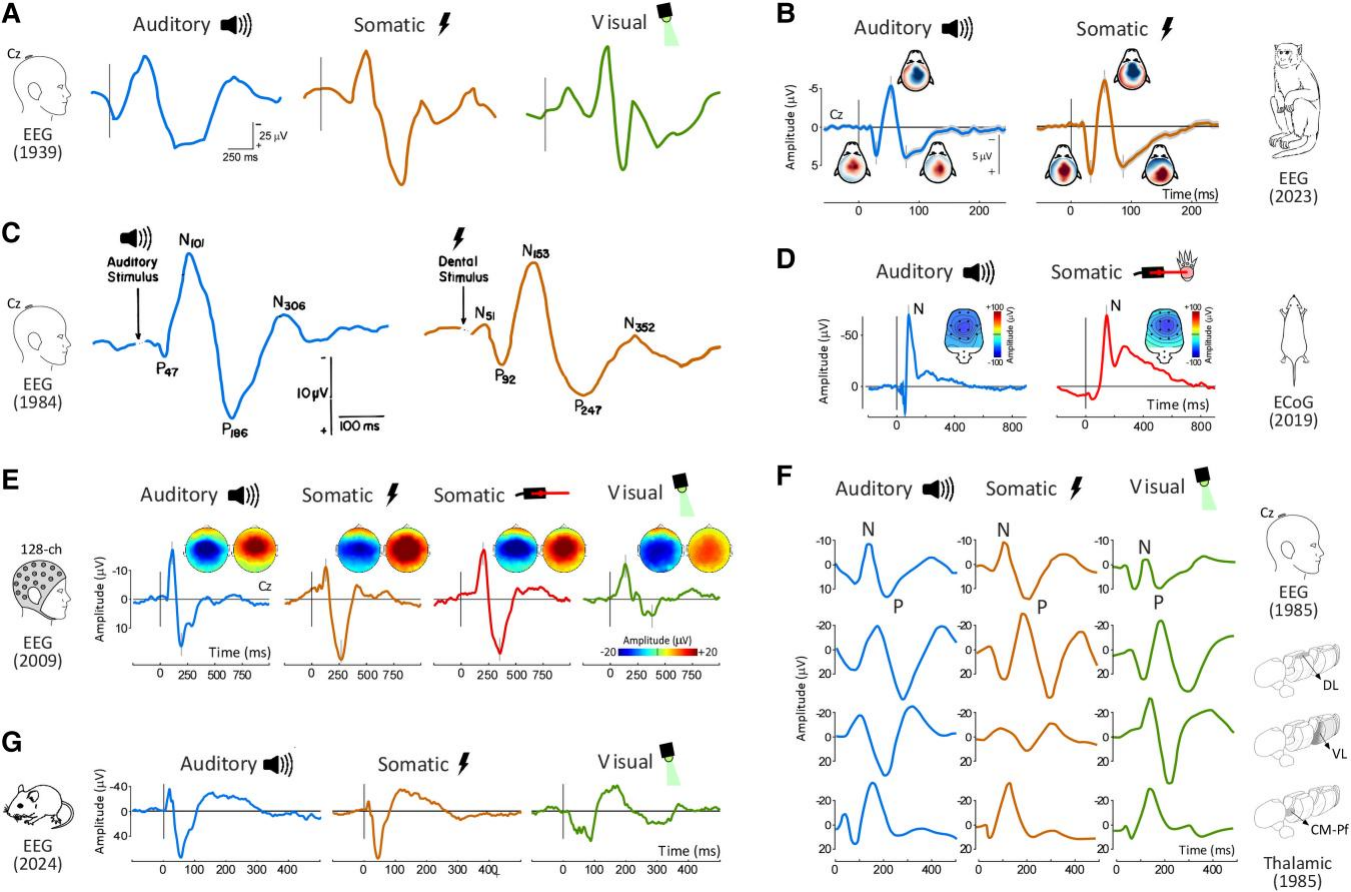
**Sudden and isolated stimuli elicit supramodal electrophysiological responses from the mammalian brain.** Sudden and isolated stimuli of different sensory modalities elicit highly similar, large and widespread brain responses, dominated by a supramodal component maximal at the scalp vertex (the vertex potential, VP). (**A** and **C**) Early work with single-channel EEG recordings first suggested that SISS in the auditory, somatosensory and visual modalities elicit highly similar electrocortical transients (adapted with permission from Davis *et al*.^5^ and Chapman *et al.*,^6^ respectively). (**E**) Later work with high-density EEG demonstrated that these responses also have highly similar scalp distributions across modalities (data from Mouraux and Iannetti^7^). (**G**, **B** and **D**) Such supramodal responses are also recorded in rat electrocorticography (data from Hu and Iannetti^8^) and in mouse (adapted from Benusiglio and Asari^9^) and monkey (unpublished data) EEG. (**F**) In simultaneous scalp and invasive recordings from human patients, similarly supramodal transients can be recorded both at the scalp vertex and in non-specific thalamic nuclei (adapted with permission from Velasco *et al.*^10^). CM/Pf = centromedian and parafascicular nuclei; DL = dorsolateral nucleus; ECoG = electrocorticography; VL = ventrolateral nucleus.

SISS elicit large and widespread cortical responses in mammals, measurable using invasive and non-invasive electrophysiology (Fig. 1; see the ‘Cortical responses to sudden stimuli: phenomenology and modality-specific interpretation’ section for a detailed description of these responses), as well as blood-flow based neuroimaging.^2,3,7,10-23^ The fact that these responses are phenomenologically similar across different mammals suggests their importance for survival.^2,8,15,19^

The main issue we address in this review is that the responses elicited by SISS are often used to study modality-specific processing in the CNS, with the implicit assumption that they are mediated by the canonical sensory pathways that project from specific thalamic relay nuclei to their corresponding primary sensory cortex.^24-39^ This issue is timely due to the persistent and widespread use of SISS in basic science studies published in high-profile journals. Many of these studies describe stimulus-evoked activity in ‘incongruent’ sensory cortices and attribute it to cortico-cortical connections or motor activity.^40-43^ However, multiple lines of evidence from humans and other animals clearly contradict this narrative. A large and surprisingly neglected body of evidence indicates that the largest component of these responses, the vertex potential (VP; not to be confused with the spontaneous ‘vertex sharp wave’ observed during non-REM sleep^44^), instead reflects the activity of a diffuse ‘extralemniscal’ thalamocortical sensory system that operates in parallel to the canonical ‘lemniscal’ sensory pathways. In this article, we critically assess this evidence and articulate a ‘two-system’ theory of sensory-evoked brain responses. We then discuss the implications of this theory to basic and clinical neuroscience, including research on the neural correlates of consciousness. Finally, we propose a novel hypothesis about the function of these surprise-related responses.

## The lemniscal and extralemniscal systems

**Figure 2:**
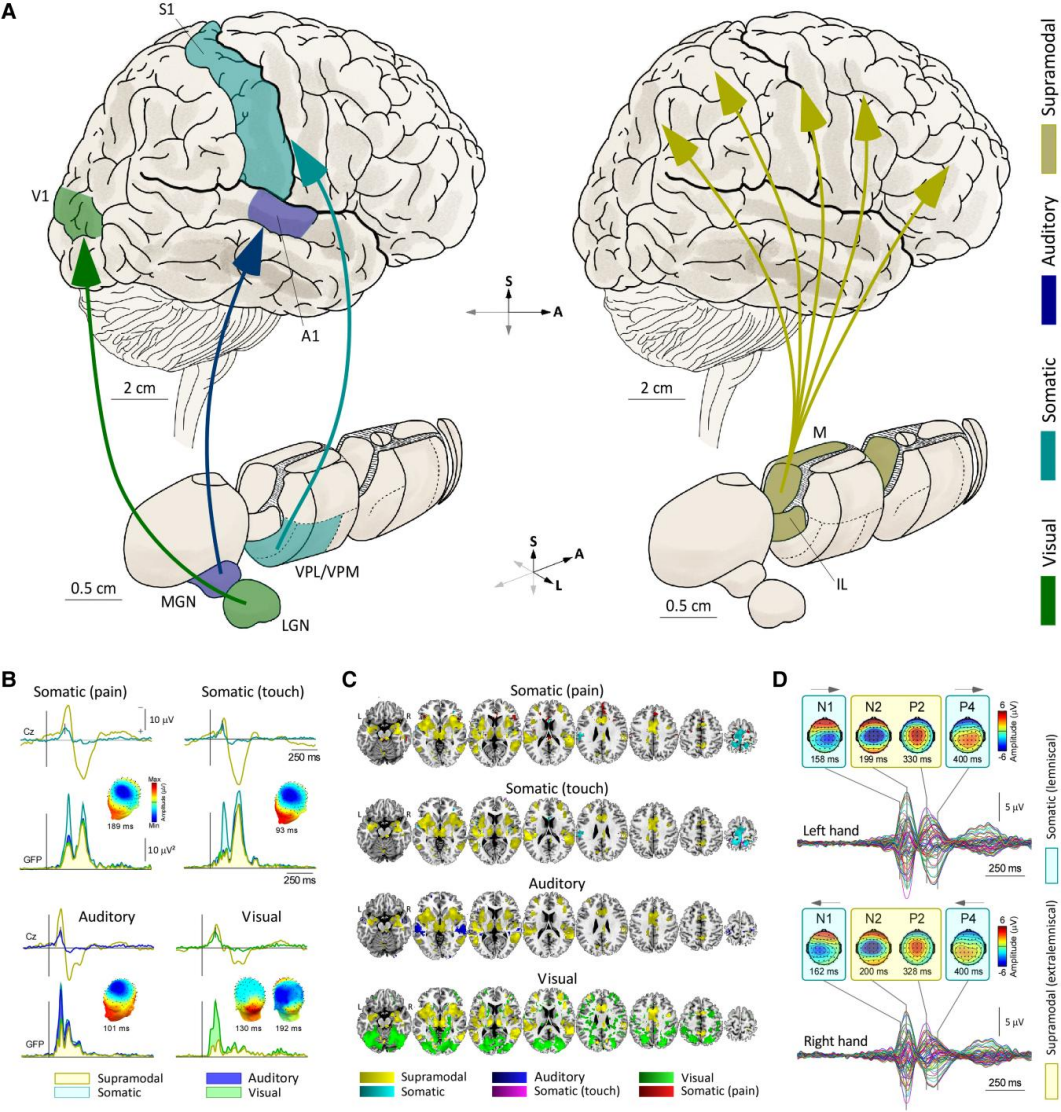
**The lemniscal and extralemniscal systems: thalamocortical projections and relative contributions to transient sensory brain responses**. (**A**) Lemniscal (specific) pathways carry high-fidelity sensory information from modality-specific relay nuclei in the thalamus to their corresponding primary sensory cortices. For example, the somatosensory lemniscal pathways (turquoise) project from the ventro-posterior nuclei (VPL, VPM) to the primary somatosensory cortex (S1). In contrast, extralemniscal (non-specific) pathways engaged by rapid stimulus changes (Fig. 3) and from non-specific intralaminar (IL) and midline nuclei (M) have widespread cortical projections (yellow). (**B**) EEG responses to sudden and isolated sensory stimuli (SISS) of four modalities are decomposed using probabilistic independent component analysis (pICA, data from Mouraux and Iannetti^7^). The obtained independent components reflect the activity of two parallel ascending sensory systems: (i) a large and widespread supramodal component (the vertex potential, yellow), reflecting the extralemniscal pathways; and (ii) smaller, spatially-restricted modality-specific components (non-yellow colours), reflecting lemniscal pathways. For each stimulus modality, the *top* waveforms show the time course of supramodal (yellow) and modality-specific (turquoise: somatic, blue: auditory, green: visual) components at the vertex electrode (Cz), whereas the bottom waveforms show the time course of GFP (global field power; standard deviation across electrodes). Scalp maps show the topographical distribution of modality-specific components. (**C**) Functional MRI responses to the same SISS (data from Mouraux *et al*.^45^) also reflect the distinction between lemniscal and extralemniscal systems. Most of the response is spatially overlapping across modalities and reflects the engagement of extralemniscal pathways. Conversely, smaller areas of activation, mostly non-overlapping and localized in primary sensory regions, reflect the engagement of lemniscal pathways. (**D**) EEG responses to somatic stimuli delivered to the left or right hand (data from Hu *et al*.^46^). The distinct and parallel activity of the two systems is noticeable even in raw EEG: we argue that the activation of the lemniscal system manifests as early N1 and late P4 peaks contralateral to the stimulated hand, reflecting the somatic input to the contralateral S1, while the extralemniscal activation manifests as the centrally-distributed vertex potential peaks (N2, P2). LGN = lateral geniculate nucleus; MGN = medial geniculate nucleus; N = negative wave; P = positive wave.

‘Lemniscal system’ is a name for the canonical auditory and somatosensory pathways that transmit environmental information from the peripheral sensory organs to the granular layer of the corresponding primary sensory cortex via thalamic relay nuclei [i.e. the medial geniculate nucleus (MGN) and the ventroposterior nuclei (VPL and VPM); Fig. 2A]. The term derives from the Greek word ‘λημνίσκος’, which means ribbon and is used to indicate a bundle of nerve fibres. (Specifically, for somatosensation: the ‘spinal lemniscus’ is composed of the lateral spino-thalamic and spino-tectal pathways, conveying information about thermal and noxious stimuli to the VPL thalamic nucleus; the ‘medial lemniscus’ is composed of the bulbo-thalamic pathways, conveying tactile information to the VPL thalamic nucleus; and the ‘trigeminal lemniscus’ conveys tactile and thermal information from the face to the VPM thalamic nucleus. For audition: the ‘lateral lemniscus’ conveys acoustic information from the cochlear nucleus to the inferior colliculi and the MGN.) Although defined anatomically in the somatosensory and auditory modalities, we extend the term ‘lemniscal system’ to refer also to the visual pathways from the retina to visual cortex via the lateral geniculate nucleus.

Lemniscal pathways are modality-specific and carry high-fidelity sensory information that allows detailed perception of the environment. For example, cells in the MGN (the thalamic relay nucleus for the auditory lemniscal pathways) display a clear tonotopy, building a population code that represents the spectral content of a sound.^47^ Similarly, the somatosensory lemniscal system comprises cells with precise somatotopic and frequency specificity (e.g. responding to flutter of a certain frequency in a limited skin area).^48,49^ Given their ability to faithfully encode a continuous sensory input, lemniscal responses are highly resistant to habituation, even when stimuli are repeated at short intervals.^50,51^ Understandably, these pathways have been a major research focus due to their importance in explaining perception.

The name ‘extralemniscal system’ refers to a set of sensory pathways parallel to the modality-specific lemniscal pathways. It was coined by French *et al*.^52^ in 1953, who described both earlier and their own results showing that under certain conditions somatosensory and auditory stimuli elicited ‘secondary’ cortical responses that were diffuse rather than confined to the primary somatosensory or auditory cortex of primates.^52,53^ These diffuse responses had distinctly longer latencies and waveforms compared to lemniscal ‘primary’ responses. Since then, a wealth of invasive recordings in rodents and cats showed similar long-latency responses in the reticular formation as well as in ‘non-specific’ intralaminar and midline thalamic nuclei that project widely to supragranular layers of the cerebral cortex (possibly via calbindin-positive matrix cells, see the ‘Are thalamic matrix cells the substrate of non-specific cortical responses? It’s complicated’ section) bypassing the primary sensory relay nuclei (Fig. 2A).^50^ Because of the similarity of these extralemniscal responses in the brainstem reticular formation and the medial thalamus, the latter structure is often considered to be the most rostral part of the reticular formation.^54^ Note that although some authors use the term ‘non-lemniscal system’ to describe multisensory cells in parts of thalamic relay nuclei that project to secondary sensory cortices,^55^ here we use the term ‘extralemniscal’ only to refer to the wider-reaching outflow from intralaminar and midline nuclei.

In stark contrast to the lemniscal system, the extralemniscal system is supramodal, i.e. it can be engaged by stimuli of several sensory modalities.^50^ This loss of identity of the modality of the afferent signal is consequent to the convergence of inputs of different modalities already at the level of the reticular formation and non-specific thalamic nuclei.^52,56^ Thus, stimuli of virtually all sensory modalities (i.e. somatic, auditory, visual and visceral) are effective in engaging the extralemniscal system, provided that they are sufficiently fast-rising and surprising.^57-59^ Extralemniscal responses habituate dramatically when stimuli are repeated at predictable inter-stimulus intervals shorter than approximately 2 s.^50,57,58,60,61^ Aside from this sensitivity to sudden changes, the extralemniscal responses in thalamus are relatively insensitive to other stimulus features, lacking the precise tonotopic or topographic specificity of their lemniscal counterparts.^50^

Despite this rich history of investigations, the extralemniscal system is nowadays neglected and largely absent from major neuroscience textbooks.^62^ This is remarkable, given that it represents a major sensory outflow of the thalamus, and likely subserves a large part of the brain responses measured in contemporary neuroscience studies.

## Cortical responses to sudden stimuli: phenomenology and modality-specific interpretation

The earliest descriptions of surprise-related responses in the human brain date back to the 1930s.^5^ Davis *et al*.^5^ showed that SISS of different modalities elicit highly similar large electrocortical transients maximal at the vertex (Fig. 1). This transient and biphasic response consists of a negative wave (N) followed by a slightly longer-lasting positive wave (P). Twenty years later, Bancaud *et al*.^63^ and Gastaut^64^ provided the first description of their scalp distribution. Having observed that the maximal amplitude of the response was measured at the vertex electrode (Cz), with a gradual fall-off symmetrical on both hemispheres, they coined the term vertex potential (VP) that is still currently used.^63,64^ In more recent years, this central and symmetrical topography was repeatedly confirmed with high-density EEG technology.^2,3,7,11-14,16,65-68^ Furthermore, it became clear that the VP occurs concomitantly to other, modality-specific components reflecting activity in the pertinent primary sensory cortex (Fig. 2B and D).^46,69^

Responses similar to the VP have also been observed with invasive recordings from cortical and subcortical structures of rodents,^2,8,19,70,71^ cats,^72^ monkeys^15^ and humans.^10,20,21^ Some of these investigations also confirmed the invariance of this response with respect to stimulus modality or stimulus location.^8,10,19-21,72,73^

When brain activity is sampled with functional MRI (fMRI), the same stimuli that evoke a VP also elicit a large and bilateral blood oxygen level-dependent (BOLD) response widespread in both cortical and subcortical structures, as well as the primary sensory cortex pertinent to the modality of the applied stimulus.^45,74,75^

The use of EEG and fMRI responses elicited by SISS to study the cortical processing of a single sensory modality is a common practice in neuroscience. These responses, despite being strongly contributed to by non–modality-specific sensory pathways, are still referred to using labels that hint, or even strongly imply, that the underlying neural activity reflects modality-specific cortical processing, exclusively or primarily mediated by the canonical lemniscal pathways.^24-39^

More importantly than the labels used, the interpretation of the recorded responses is often limited to the modality of the applied stimulus or the function investigated. The pain field offers glaring examples of this incorrect reasoning. The idea that the EEG or fMRI responses elicited by sudden and isolated nociceptive stimuli causing pain reflect the activity of a ‘pain matrix’ or a ‘pain signature’ has been hegemonic for a long time (for a detailed discussion, see Hu and Iannetti^76^ and Mouraux and Iannetti^77^). Despite an ongoing debate about the specificity of these responses, statements like ‘The pain matrix is thought to play a key role in elaborating two important aspects of the nociceptive experience: the sensory-discriminative aspect and the affective-motivational aspect’^78^ or ‘pain matrix; a network of brain regions that is activated in response to nociceptive stimuli and contributes to pain perception’,^79^ remain pervasive. Similarly, the electrocortical responses elicited by noxious laser stimulation are interpreted by many (including ourselves in our initial publications) as reflecting nociceptive processing and pain perception.^26,29-31,80,81^ Other examples come from auditory neuroscience research, where the responses elicited by auditory SISS are often interpreted as a measure of neural activity in auditory cortex, and used to infer neural mechanisms subserving hearing.^28,33,34,36,39,82-85^ For example, Wagner *et al*.^36^ state that ‘Neural ensembles within auditory cortex respond to acoustic features within the spoken word. These cortical responses are reflected within the P1-N1-P2 and T-complex waveforms of the auditory evoked potentials (AEP)’. The issue is that, while technically true, statements like this downplay the possibility that the P1-N1-P2 response is largely contributed to by non–modality-specific activity outside of auditory cortex. Similarly, the ‘acoustic change complex’ (ACC)^25^ is defined as a ‘negative–positive complex that is elicited by a change occurring during an ongoing acoustic stimulus’^32^ and is often interpreted in an auditory-specific fashion, for example, ‘The ACC indicates the encoding of potentially discriminable information at the level of the auditory cortex’.^86^ However, our own previous work demonstrated that such abrupt changes during ongoing stimuli elicit a VP practically identical to that elicited by an impulsive stimulus.^2,3^ Given that the ACC paradigm is often used in clinical work to study (lemniscal) auditory processing,^87-89^ we have already warned against the use of this term and the modality-specific functional interpretations that often accompany it.^2,3^

These modality-specific interpretations are often justified by source analysis studies that found neural generators in sensory regions pertinent to the modality of the eliciting stimulus.^35,38,90-93^ However, source analysis of EEG has a very high degree of uncertainty due to the extremely large solution space and is highly prone to confirmation bias, given that source analysis algorithms often require prior assumptions about the number and initial locations of equivalent dipoles.^94^ This problem is compounded when the analysis is applied to a widespread EEG response reflecting the concomitant activity of many neural sources,^94^ as is the case for the responses elicited by SISS,^4,7,70,71^ as well as the following section.

It is not far-fetched to say that most sensory evoked potential studies do not mention the possibility that the response under examination at least partly reflects non–modality-specific brain activity. This important neglect, together with the widespread use of modality-specific labels and interpretations of responses that are in fact largely supramodal, obstructs understanding of both physiological and pathological studies, and thereby could misinform future clinical decisions.^95^ This issue is a special case of the more general problem of dissecting local versus global neural activity when interpreting the nature of cortical processing. For example, when aiming to explore task-related neural dynamics, spontaneous, non-task related movements result in global cortical activity, and represent potentially serious confounds in cognitive neurophysiology.^96^

## Cortical responses to sudden stimuli are largely comprised of non-specific extralemniscal activity

The core message of this paper is that the cortical responses elicited by the large majority of sensory stimuli used in cognitive neuroscience are composed of distinct components reflecting the relative contribution of the lemniscal and extralemniscal pathways. We call this view the ‘two-system’ theory of sensory evoked brain responses. Importantly, we are not claiming to describe any novel physiology, as the extralemniscal system has a rich history of investigations and for many years was regularly taught in textbooks.^50,97^ Rather, the two-system theory describes the contribution of this known physiology to commonly recorded stimulus-evoked neural responses. Spelling out this contribution is important given that the extralemniscal system is nowadays neglected^62^ and brain responses are often interpreted in a modality-specific fashion.

As we will discuss later, the relative contribution of these two systems is highly variable, mostly depending on several bottom-up stimulus properties but also on the context in which stimuli are delivered. Importantly, in the case of sudden and isolated sensory stimuli delivered at inter-stimulus intervals longer than some seconds (i.e. SISS), which are extremely common in cognitive and sensory neuroscience research, the contribution of the extralemniscal system dominates. Indeed, as detailed in the previous section, while fMRI BOLD activity elicited by SISS of several modalities does reveal a local activation of the primary sensory cortex pertinent to the modality of the eliciting stimulus, it also reveals a much larger, global cortical activation common across different stimulus modalities (Fig. 2C).^45^ These local and global activities likely reflect the engagement of lemniscal and extralemniscal thalamocortical projections, respectively. Also, dynamic causal modelling of this set of BOLD responses demonstrates that the widespread cortical activation is mediated by extralemniscal thalamocortical pathways that bypass the primary sensory cortices, whereas the local cortical activation is mediated by lemniscal thalamocortical pathways projecting directly to the primary sensory areas.^18^

In the case of scalp electrophysiology, the cortical origins of sensory-evoked potentials are less clear. Still, its high temporal resolution provides a helpful tool to pin down the relative contributions of lemniscal and extralemniscal pathway activity. Later, we review the evidence that the neural activity consequent to the engagement of the extralemniscal system dominates the EEG response when it is elicited by SISS.

### Phenomenological similarity of the EEG response across modalities

The first compelling piece of evidence is the qualitative similarity of the cortical responses elicited by SISS belonging to different modalities, with respect to both the polarity of their components and their scalp distribution.^6,7,10,20,63^ A defining property of the extralemniscal system is its supramodality, as shown by the similarity of response morphology in non-specific thalamus across sensory modalities.^20,50^ Thus, the repeated observation that the scalp response is highly spatially similar across sensory modalities suggests that it largely reflects extralemniscal activity, with minimal contribution of modality-specific lemniscal pathways.

Beyond initial qualitative comparisons of scalp topographies,^63^ more recent studies have used quantitative methods to dissect the constituent components of the response. These methods range from simple EEG re-referencing to isolate local from global components^26,98^ to more sophisticated techniques such as adaptive spatial filtering,^99^ microstate analysis^46^ and probabilistic independent component analysis.^7,100^ All these approaches have revealed that the EEG response to SISS is dominated by a large and global supramodal subcomponent (the VP), while more local and modality-specific subcomponents offer a smaller contribution (Fig. 2B and D).^7,46,100^ For example, the response to somatosensory SISS consists of the N1 and P4 components, which contribute to the earliest and latest part of the response and reflect neural activity in the corresponding primary sensory cortex contralateral to the stimulated body part, and the vertex N and P components, which dominate the middle part of the response and reflect activity arising from more diffuse, bilateral cortical generators (Fig. 2D).^46,69,100^ Furthermore, the trial-by-trial latencies of the modality-specific N1 and P4 components correlate with each other, but not with the vertex N and P waves (and vice versa),^46^ providing further evidence that the N1 and P4 components (on the one side) and the vertex N-P complex (on the other side) reflect distinct, parallel systems.

Animal literature is also informative in this respect. For example, invasive recordings in cats reveal similar responses across sensory modalities in several widespread cortical areas, nicely dovetailing what is observed in human scalp electrophysiology.^72,101^ It is interesting to note that in this animal literature, those widespread supramodal cortical responses were never interpreted as reflecting modality-specific lemniscal processing.

### Sensitivity to sudden sensory changes

**Figure 3:**
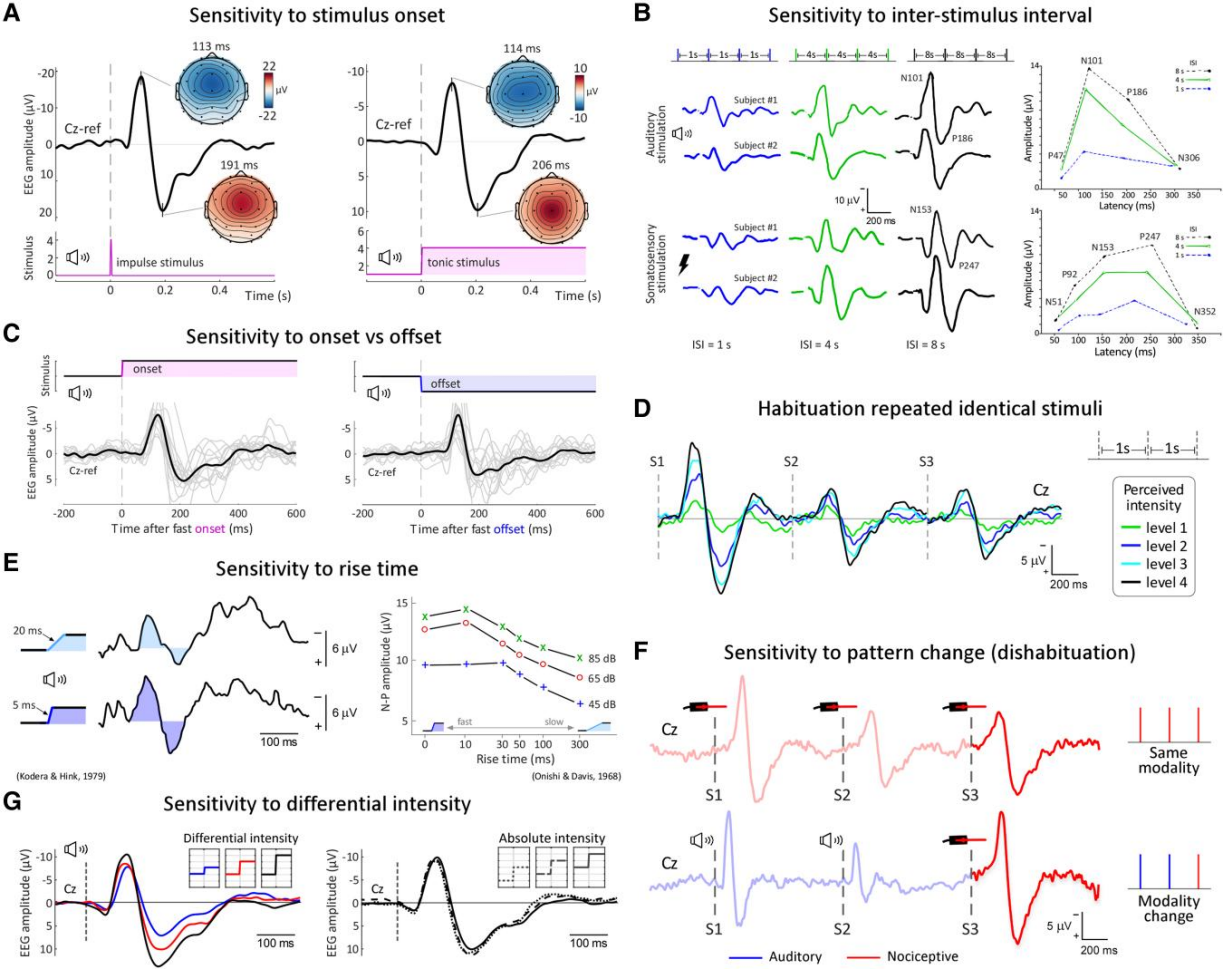
**Vertex potential magnitude is sensitive to low-level stimulus features that determine its surprise content.** The vertex potential (VP) is effectively elicited by sudden changes of stimulus energy, regardless of whether after a fast onset the stimulus returns to baseline (**A**, *left*) or persists tonically (**A**, *right*, data from Somervail *et al*.^2^). Rapid offsets also elicit the VP response (**C**, data from Somervail *et al.*^3^). VP magnitude is sensitive to the rise time of stimulus changes (**E**, adapted with permission from Kodera and Hink^102^ and Onishi and Davis^103^). VP magnitude is also sensitive to the differential, but not absolute, stimulus intensity (**G**, data from Somervail *et al*.^2^). **A**, **C**, **E** and **G** show that the VP magnitude is determined by the rate of change of stimulus energy. Besides how the stimulus changes with respect to the immediately preceding baseline, the VP magnitude is also largely determined by the properties of the pattern of preceding stimuli: for example, longer inter-stimulus intervals (ISIs) result in larger VP responses (**B**, adapted with permission from Chapman *et al.*^6^). Crucially, this effect is only present for the mid latency VP peaks that we argue reflect the extralemniscal system, while it does not affect the earliest and latest peaks that likely reflect the modality-specific lemniscal system (**B**, *right*; see also Fig. 2D). This observation dovetails the tendency of the extralemniscal system to habituate to repeated stimulation, and the ability of the lemniscal system to faithfully encode stimuli delivered at higher frequencies. The VP habituation is strongest when stimuli are delivered at a short and constant ISI (e.g. 1 s, **D**, data from Iannetti *et al.*^104^). This effect disrupts the correlation between VP magnitude and perceived stimulus intensity. A change in one of the fundamental features defining every sensory stimulus, such as its modality, will reverse the VP habituation (**F**, data from Valentini *et al.*^13^). Stimuli with the features summarized here can be described as ‘sudden and isolated sensory stimuli’.

Human EEG studies have clearly shown that the amplitude of the VP is highly sensitive to both stimulus rise-time^102,103^ (Fig. 3E) and the amount of stimulus intensity change^2^ (Fig. 3G): when fast-changing and intense stimuli are used, whether increases or decreases of intensity (Fig. 3A and C), the resulting EEG responses are largely composed of the VP component. This sensitivity to sudden changes parallels another defining property of the extralemniscal system: the fact that it is only engaged by fast-rising stimuli (e.g. SISS), whereas the lemniscal system also faithfully encodes slow-rising and tonic stimuli, for example, by slowly applied pressure, light touch or hair bending.^50,57,58^

### Habituation to repeated stimulation

A third piece of evidence indicating that extralemniscal neural activity dominates the EEG response to SISS is the fact that the bulk of the response habituates when the stimulus is repeated at short and predictable intervals (i.e. when the stimulus is no longer isolated; Fig. 3B, D and F).^6,11,104-108^ This observation indicates that the response does not primarily reflect the activation of the lemniscal system, which reliably responds to stimuli repeated as fast as 10 Hz, but rather the extralemniscal system, which is optimally engaged by isolated stimuli.^50,57,58,60,61^ Consider, for example, that to obtain a clean readout of lemniscal somatosensory processing when measuring the N20-wave elicited by electrical stimulation of the median nerve, high frequencies of stimulation (up to 10 Hz) are recommended.^109^

The difference in habituation of the lemniscal and extralemniscal responses to repeated stimulation provides elegant evidence for the two main messages of this work: that sensory-evoked brain responses reflect a mixture of lemniscal and extralemniscal activities, and that when sudden stimuli are presented in isolation, the extralemniscal activity can dwarf the lemniscal activity. For instance, Chapman *et al*.^6^ showed that increasing the frequency of stimulation dramatically dampens the large supramodal VP component of the response evoked by either auditory or somatosensory stimuli, while leaving earlier and later modality-specific components largely unaffected (Fig. 3B).

### Lesion studies

Perhaps the strongest causal evidence that the cortical responses elicited by SISS largely reflect the activation of the extralemniscal system comes from lesion studies in animal models. Indeed, both ablation and pharmacological inactivation of modality-specific lemniscal thalamic nuclei or primary sensory cortical areas leave the vertex response largely unaffected.^70-72,110^ Even when larger cortical territories surrounding the primary cortices are removed, a full-fledged VP is observed.^72,110^ Similarly, the VP elicited by auditory stimuli is mostly unaffected by ischaemic damage to auditory cortex in human patients.^106,111^ Notably, even when the lemniscal response in primary cortices is enhanced by strychnine, again the non-specific diffuse response is unaffected.^110^ Altogether, this large and sadly neglected body of empirical evidence (e.g. the extremely detailed and informative experiments of Buser and Borenstein^110^ have been cited, at the time of publication of the present work, only 39 times) shows a clear independence of the cortical responses evoked by the engagement of extralemniscal versus lemniscal pathways.

### Sensitivity to anaesthesia

Lemniscal activity in both modality-specific thalamic nuclei and primary sensory cortices is remarkably preserved during anaesthesia. In contrast, the extralemniscal system is particularly sensitive to anaesthesia: responses in non-specific thalamic nuclei and their widespread cortical projections are selectively abolished by most general anaesthetics, with the notable exclusion of chloralose.^50^ Similarly, general anaesthetics completely abolish the widespread VP component, while leaving the early-latency primary sensory cortical components intact.^70,72^ Similar results have been found in humans, with VPs being attenuated by general anaesthetics such as propofol,^112^ midazolam,^113^ alcohol^79^ and nitrous oxide.^114^

Together with the ablations of the primary auditory cortex described in the previous paragraph, the results of Simpson and Knight^71^ in particular show an exquisite double-dissociation of the contribution of the lemniscal and extralemniscal systems to the epidural response: barbiturate anaesthesia selectively abolishes the vertex response with no effect on the early (auditory-cortical) components, while selective ablation of the auditory cortex abolishes these early components, while leaving the vertex-response unaffected.^70,71^

### Thalamic stimulation

Additional causal evidence comes from direct intrathalamic stimulation: the same isolated electrical stimulation in both cats and monkeys elicits dramatically different cortical responses depending on the targeted thalamic nuclei.^115^ The stimulation of somatosensory-specific lateral thalamus evokes small-amplitude cortical responses likely to pass unnoticed as they are restricted to the contralateral primary somatosensory cortex. In striking contrast, stimulation delivered only a few millimetres more medially, in the intralaminar nuclei, evokes large-amplitude responses widespread across the cortex.^115^ Clearly, this result provides further evidence that the widespread, symmetrical distribution of the VP cannot be explained by the lemniscal pathways.

## Are thalamic matrix cells the substrate of non-specific cortical responses? It’s complicated

The classical concept of the extralemniscal system was based on anatomical and electrophysiological observations about ‘non-specific’ thalamic nuclei such as the intralaminar and midline nuclei.^50,52,116^ However, early electrophysiological recordings were blind to specific cell types, and recent work has revealed a substantial diversity in thalamocortical cells: for example, cells located in these intralaminar and midline nuclei have been shown to have distinct afferent and efferent connectivity patterns, even when located within the same nucleus.^117,118^ Thus, the concept of functionally-homogenous ‘non-specific’ thalamic nuclei, initially supported by electrophysiological evidence,^50,115^ has gone out of fashion.^119-121^ This clearly highlights the importance of a cellular understanding of thalamocortical systems.

Considering the distinction between ‘matrix’ and ‘core’ cells proposed by Jones^116,122,123^ is particularly relevant for this discourse. Parvalbumin-positive core cells dominate in specific sensory and motor relay nuclei (although they can also be found in intralaminar nuclei) and project to limited cortical areas, synapsing at middle layers.^116,122,123^ In contrast, calbindin-positive matrix cells are found throughout the thalamus and project widely across the cortex, synapsing at superficial layers. Similar to non-specific nuclei, matrix cells have broad receptive fields resulting in low-fidelity representations of sensory stimuli, for example, low tonotopic specificity in audition and low somatotopic specificity in somatosensation.^50,116,123^ Jones argues that the non-specific integrative functions ascribed to the intralaminar and midline nuclei are in fact subserved by matrix cells,^116,123^ which generalizes the concept of ‘non-specific thalamus’ beyond the intralaminar and midline nuclei. Owing to these similarities, we have previously related supramodal responses to matrix cells.^124^ However, there remain several issues with this account.

First, although matrix cells are found throughout the thalamus, local field potential recordings from various thalamic structures containing matrix cells do not show VP-like responses. For example, there are matrix cells which respond to stimuli of multiple modalities in the vicinity of the somatosensory-specific ventroposterior nucleus (VPL) and of the auditory-specific MGN,^55,116^ but electrophysiological recordings from these areas do not show VP-like responses.^10^ Indeed, these matrix cells only project to the cortical area surrounding the primary sensory cortex corresponding to the thalamic relay nucleus core, which they surround,^116^ making it unclear how they could account for the practically identical widespread scalp topographies elicited by SISS of different sensory modalities.

Second, the non-specific centromedian nucleus (CM) is almost entirely devoid of matrix cells,^116,125^ although it also shows several physiological properties that make it a compelling candidate substrate for the VP. Indeed, (i) recordings from the CM reveal a rapid habituation to repeated identical stimuli; (ii) CM inactivation suppresses supramodal cortical responses; and (iii) CM stimulation produces a widespread VP-like scalp response.^10,57,58,72,126,127^

Third, mapping of the cortical regions whose BOLD signal correlates with matrix activity suggests that the matrix projections are somewhat asymmetric along the midline,^125^ in clear contrast to the symmetrical VP topography.^7,100^

Owing to these issues, we remain sceptical that matrix cells are the substrate of the non-specific cortical response elicited by SISS. Not surprisingly, the matrix-core dichotomy has been suggested to be simplistic, and calbindin-positive cells are not the only thalamocortical cells with diffuse projections to cortex.^117^ Calbindin-positive cells may also be functionally heterogenous, given the fact that their projections are still constrained by the thalamic nucleus of their origin.^116^ In addition, a population of core cells located in the intralaminar and midline thalamic nuclei, each with a limited cortical target but projecting, at population level, to a range of cortical areas could also explain a widespread EEG response such as the VP. Future work could tackle the important question of whether thalamic matrix cells contribute to the VP measured in scalp EEG.

## Further implications of the two-system theory for basic and clinical neuroscience studies

The two-system theory we have outlined has major implications for interpreting brain responses across a wide range of basic and clinical neuroscience studies. In this section, we provide additional examples from other fields of investigation, and conclude with recommendations for distinguishing lemniscal from extralemniscal components in the brain responses evoked by sensory stimuli.

### Implications for studies of the neural correlates of consciousness

The logic of the two-system theory is highly relevant to current debates on the specificity of the neural correlates of consciousness (NCCs), particularly in studies of conscious content using SISS. Widespread, supramodal responses to such stimuli (like the P3 in EEG studies) have been interpreted as signatures of conscious awareness.^128-131^ However, similarly to the ‘pain matrix’ debate detailed in the ‘Cortical responses to sudden stimuli: phenomenology and modality-specific interpretation’ section, this assumption is questionable, as explained later.

In typical NCC paradigms, low intensity, near-threshold SISS are used, and the brain activity measured in trials in which the stimulus was perceived is contrasted to that measured in non-perceived trials. In EEG studies, this contrast often highlights a transient widespread response (often referred to as P3 or P300), which has been, perhaps too hastily, interpreted as reflecting conscious awareness.^128-131^

The P3 shares many similarities with the VP: it has a widespread scalp topography,^128,132,133^ it is elicited by sudden stimuli regardless of their sensory modality,^132-136^ and it is sensitive to stimulus unexpectedness^133,136^ (e.g. in ‘oddball’ designs, a behaviour highly reminiscent of the VP dishabituation effect shown in Fig. 3F) and task-relevance.^134,137-141^ Therefore, the transient P3 reported in NCC studies likely also reflects extralemniscal activity.

It follows that the engagement of the extralemniscal system described in this article can be a fundamental confound not only when studying modality-specific sensory processing, but also when studying the NCCs with protocols involving sudden, isolated (e.g. >2 s apart) or rare stimuli. Indeed, when consciousness is probed with longer lasting images containing faces which are either perceived or not, depending on whether participants are primed to detect it, the conscious perception is not encoded by transient responses, but only by a sustained occipital-temporal negativity.^142^ Similarly, controlling for task-relevance abolishes the P3.^134,137,141^ Such results cast serious doubt on claims that transient, widespread EEG responses such as the VP and P3 are genuine NCCs.

A factor further complicating the picture is that extralemniscal EEG components are highly sensitive to anaesthesia—a manipulation commonly used in NCC research. Both extralemniscal activity and conscious awareness are abolished by most anaesthetics. However, this correlation is not obligatory and can be dissociated: The anaesthetic chloralose, for example, enhances extralemniscal responses while abolishing consciousness.^50,70,71^

Because both extralemniscal activity and consciousness are sensitive to many of the same manipulations, their co-occurrence in NCC paradigms can misleadingly suggest a direct causal link. This perspective is reminiscent of previous arguments that the widespread P3 response may reflect a post-perceptual process rather than a true NCC,^131,143^ with the crucial difference that extralemniscal activation does not rely on the conscious perception of the eliciting stimulus.

In summary, researchers studying consciousness should be aware that transient sensory stimuli—including those from transcranial magnetic stimulation, a technique frequently used in consciousness studies^144^—can trigger widespread extralemniscal activity that may be mistaken for NCCs, unless they are effectively controlled for.^145,146^ This is a relevant issue given that many influential theories of consciousness posit that widespread cortical integration is itself the neural basis of consciousness,^129,147-149^ a notion that might have been prompted by the large and widespread cortical responses mistakenly interpreted as NCCs. For this reason, recognizing and controlling for this confound is essential.

### Implications for studies of sensory dysfunction

In the clinical domain, proper understanding of dysfunctional sensory systems relies on correct attribution of brain responses or statistical effects to the lemniscal or extralemniscal sensory systems. In a recent clinical study, Miyakoshi *et al*.^150^ explicitly used our two-system theory to interpret their results: they examined both lemniscal (auditory steady-state response; ASSR) and extralemniscal (VP) responses as EEG biomarkers of fragile X syndrome. Adopting this perspective allowed the authors to understand the apparently contradictory results: in fragile X syndrome patients, the VP was enhanced and the ASSR was reduced. Thus, their auditory hypersensitivity appears to arise from extralemniscal, rather than lemniscal hyperactivity. The authors also found that the VP amplitude—a readout of extralemniscal activity—has a greater sensitivity towards classifying patients with fragile X, further demonstrating the clinical utility of this theoretical framework.

### Recommendations for distinguishing lemniscal and extralemniscal neural activity

As shown earlier, widespread extralemniscal components dominate the brain response elicited by sudden, intense, and isolated stimuli, making its interpretation fairly straightforward. However, strictly speaking, the EEG response is always a mixture of lemniscal and extralemniscal components. When stimuli are less sudden, lower in intensity, or less unexpected, the interpretation of the resulting brain response becomes more ambiguous.

For this reason, we recommend examining the scalp topography of brain responses—or of any statistical effects—and quantitatively comparing^3,151^ them with the known widespread, central distribution of the extralemniscal VP versus the more spatially restricted topographies of lemniscal components, such as the somatosensory N1 or P4.^46,69^ Similar comparisons can be made when interpreting results from spatial filtering methods such as independent components analysis^7,100,152^ or generalized eigendecomposition.^153^ In addition, using multiple stimulus modalities^7,100^ and habituation paradigms^11,104,154^ is often highly informative, as these approaches test whether brain responses or extracted components display the hallmark extralemniscal properties of supramodality and sensitivity to stimulus repetition, respectively.

## What is the functional significance of the extralemniscal response to sudden sensory stimuli?

**Figure 4:**
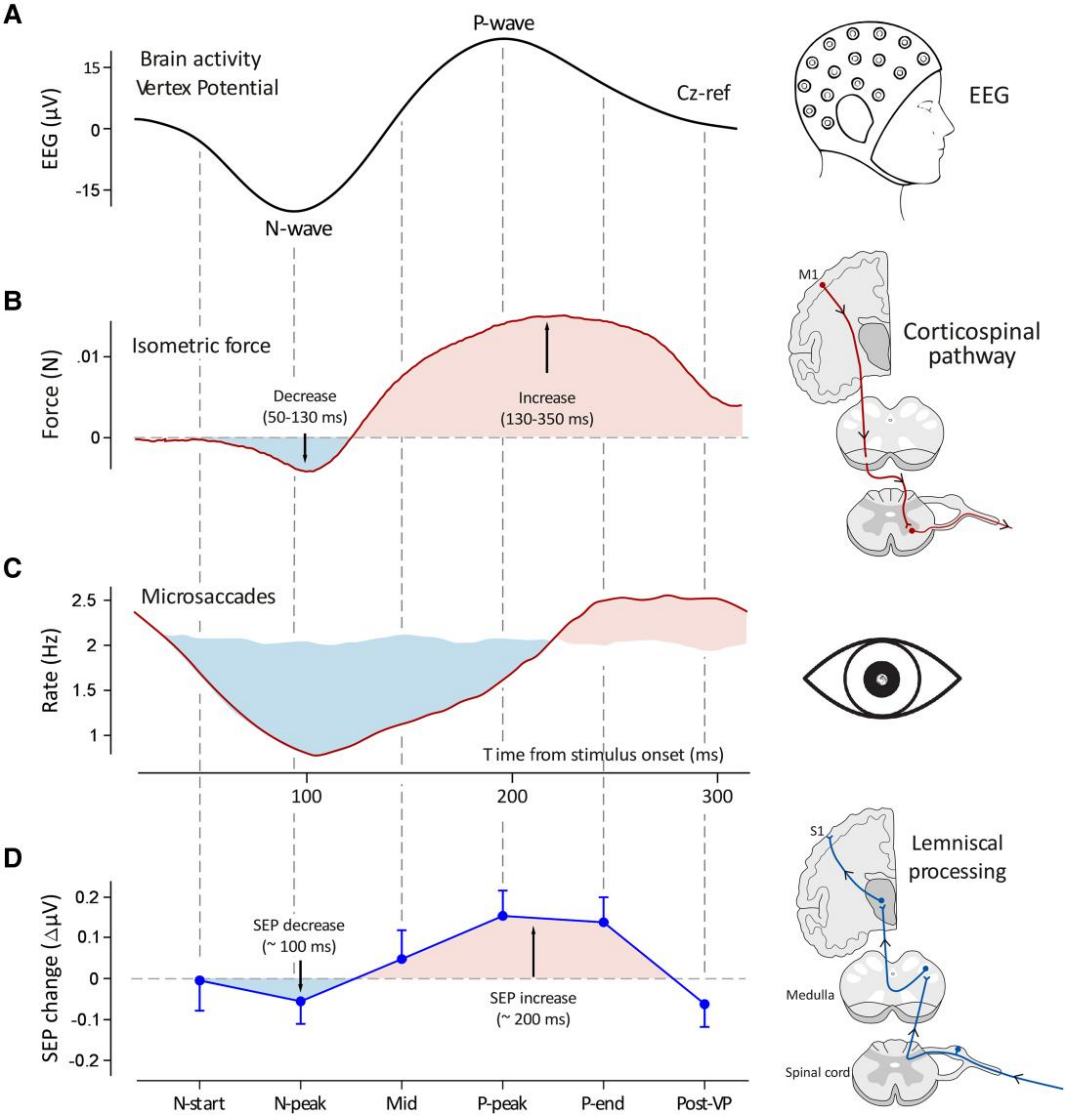
**Coupling between vertex potential occurrence and modulations of ongoing cortical function.** The main tenet of this work is that the vertex potential (VP) elicited by sudden and isolated sensory stimuli (SISS; **A**) reflects the engagement of the extralemniscal system and its widespread thalamocortical projections. This interpretation parsimoniously explains the observation that the VP is tightly linked to a modulation of ongoing cortical function across several functional domains, often taking the form of an inhibition followed by an enhancement of ongoing cortical activity (**B–D**). This bipolar modulation is observed when the corticospinal system is engaged in an isometric force task (**B**, data from Novembre *et al.*^12^) and in microsaccades (**C**, adapted with permission from Engbert and Kliegl^155^). A similar modulation is observed in the responsiveness of the primary somatosensory cortex to lemniscal input (**D**, data from Perovic *et al.*^156^).

The two-system theory also provides valuable insights into the function of the extralemniscal component of the brain response elicited by sensory events. Unlike their lemniscal counterparts, which subserve domain-specific processing of sensory information, the nuclei of the extralemniscal system are involved in domain-general modulation of the global brain state.^121^ Accordingly, there is clear evidence that the VP waveform reflects a rapid fluctuation of excitability across wide cortical territories, consisting of a decrease followed by an increase of excitability (Fig. 4). This is shown by measuring both cortical motor output,^12,155^ and cortical responsiveness to sensory input.^156^

This rapid succession of reduction and enhancement of cortical excitability is reminiscent of what occurs during sleep slow waves.^157,158^ Indeed, sleep slow waves (and K-complexes in particular), besides having a widespread, central and symmetrical scalp distribution similar to the VP recorded in wake and habituating similarly in response to high-frequency repetitive stimuli,^159^ reflect a dramatic fluctuation of membrane potential and cortical excitability^157,160^: during the negative peak of a slow wave, cortical neurons are hyperpolarised and their firing ceases (a cortical down-state). This is followed by a state of depolarization in which cells fire more readily (a cortical up-state).^157,160^ In sleep, this succession between up- and down-states repeatedly disrupts brain connectivity and consciousness,^161^ and has been conceptualized with the admittedly simplified idea of a ‘reboot’ of the brain.^157^ In wakefulness, the biphasic VP waveform may reflect a similar, albeit weaker phenomenon: a ‘cortical reset’ which interrupts the ongoing brain activity but without a dramatic impact on conscious experience.

A parsimonious hypothesis is that this cortical reset indexed by the VP would facilitate rapid task-switching by interrupting less urgent brain processes and allowing novel and potentially life-saving sensory information to be more effectively processed. It is important to highlight that under this hypothesis, a cortical reset indexed by the VP does not create an optimal behaviour itself, but rather provides a necessary substrate: an increase of cortical responsiveness to effectively and swiftly act if needed (e.g. escaping from a predator or catching prey). Accordingly, the VP is followed by a few-second long increase of typical proxy-measures of central arousal, such as pupil diameter and skin conductance.^162,163^ Crucially, the increase of these measures is predicted by the amplitude of the preceding VP.^162,163^

These effects are consistent with the idea that the VP reflects the output of a diffuse extralemniscal projection. Indeed, stimulation of non-specific thalamic nuclei results in aroused behavioural states.^164-167^ Also, stimulation of the CM nucleus in humans results in broadband increases in gamma frequency power and decreases in alpha frequency power^168^ similar to those observed following the VP.^8,169,170^

Thus, the extralemniscal component of the cortical response to sensory stimuli exerts a domain-general modulation of the brain state, which we hypothesize facilitates the interruption of ongoing lower-priority behaviours, establishes an aroused brain state, and thereby promotes swift reactions to avoid or exploit any threats or opportunities afforded by the environmental change.

This hypothesis could be empirically tested in a number of ways, by measuring whether trial-by-trial variability of VP predicts behavioural performance or certain physiological parameters reflecting the interruption of ongoing brain function, such as (i) task-switching effectiveness; (ii) performance of a working memory recall task; or (iii) persistence of a given EEG cortical rhythm entrained with tACS brain stimulation methods.

## Concluding remarks

We have presented substantial evidence that (i) the brain responses elicited by sensory stimuli reflect the relative contribution of both lemniscal and extralemniscal sensory systems; and (ii) when SISS are used, the extralemniscal contribution dominates. Although there remains some uncertainty about the exact cellular substrates of this extralemniscal component, the perspective we articulate here provides a crucial interpretative framework relevant to practically every EEG or fMRI study in basic and clinical neuroscience using sudden and isolated sensory stimuli. This framework is critical for understanding not only high-profile papers reporting sensory responses in ‘incorrect’ primary sensory areas,^40,42^ which could be more parsimoniously interpreted as reflecting non-specific thalamocortical systems, but also studies of the neural correlates of consciousness, where widespread responses to sudden, isolated, or rare stimuli—often interpreted as signatures of awareness—may instead reflect extralemniscal activity. Finally, we propose a novel and biologically plausible account of the functional significance of these extralemniscal responses: that they reflect a cortical reset that facilitates the interruption of ongoing behaviours and swift reactions to unexpected environmental changes.
